# Incorporating Blood Flow in Nerve Injury and Regeneration Assessment

**DOI:** 10.3389/fsurg.2022.862478

**Published:** 2022-04-20

**Authors:** Stewart Yeoh, Wesley S. Warner, Samer S. Merchant, Edward W. Hsu, Denes v. Agoston, Mark A. Mahan

**Affiliations:** ^1^ Department of Neurosurgery, University of Utah, Salt Lake City, Utah, United States; ^2^ Department of Biomedical Engineering, University of Utah, Salt Lake City, Utah, United States; ^3^ Department of Anatomy, Physiology, and Genetics, Uniformed Services University of the Health Sciences, Bethesda, Maryland, United States

**Keywords:** perfusion, nerve injury, regeneration, peripheral nerve, blood vessel, Sunderland injury classification

## Abstract

Peripheral nerve injury is a significant public health challenge, with limited treatment options and potential lifelong impact on function. More than just an intrinsic part of nerve anatomy, the vascular network of nerves impact regeneration, including perfusion for metabolic demands, appropriate signaling and growth factors, and structural scaffolding for Schwann cell and axonal migration. However, the established nerve injury classification paradigm proposed by Sydney Sunderland in 1951 is based solely on hierarchical disruption to gross anatomical nerve structures and lacks further information regarding the state of cellular, metabolic, or inflammatory processes that are critical in determining regenerative outcomes. This review covers the anatomical structure of nerve-associated vasculature, and describes the biological processes that makes these vessels critical to successful end-organ reinnervation after severe nerve injuries. We then propose a theoretical framework that incorporates measurements of blood vessel perfusion and inflammation to unify perspectives on all mechanisms of nerve injury.

## Introduction

Unlike the central nervous system, peripheral nerves undergo genetically defined programs for regrowth and consequent restoration of function; however, this regenerative capacity is dependent on nerve injury severity, and patient outcomes can be frustratingly inconsistent (1, 2). For the estimated 18,700 individuals annually in the United States who experience a traumatic peripheral nerve injury, a substantial fraction will endure lifelong pain or loss of function (3–5). The challenge in improving outcomes is our inadequate understanding of the key mechanisms underlying injury severity.

The classic Seddon grading scheme classifies peripheral nerve injuries based on clinical outcomes and / three grades: neurapraxia, a temporary conduction block; axonotmesis, muscular atrophy consistent with axonal discontinuity; and neurotmesis, a complete disruption of the nerve. Sunderland expanded on this system in 1951 (6) to add greater scientific and microanatomical basis and to further differentiate the highly variable recovery potential seen clinically. To expand and explain variable outcomes in axonotmetic injuries, a third-degree injury was described as internal disorganization of the endoneurium with intact perineurium, fascicular architecture, and epineurium that leads to nerve recovery; a fourth-degree injury as disruption of the perineurium and fascicular architecture with intact epineurium which does not regenerate; and fifth-degree injury as complete loss of nerve trunk continuity. Although Sunderland’s nerve injury grades provided a finer distinction, evidence for their specific mechanistic description is questionable; particularly the intact epineurium of fourth-degree injury (7).

Since Sunderland, no other grading system has been established, despite the diversity of mechanisms underlying nerve injury. Moreover, these systems lack information on both the cellular mechanisms underlying injury outcomes, and the role of nerve-associated blood vessels in regeneration. Inflammation (8–10) and ischemia (11–14) play critical roles in whether recovery from injury is successful or dysfunctional. Additionally, ischemia and inflammation are intimately connected and can lock together in feedback loops that lead to poor outcomes (15).

We seek to outline the known implications of the role of nerve-associated vasculature, as well as its interplay with the immune system in response to injury, so that this element can be considered in future conceptual models. Furthermore, we discuss the development of meaningful diagnostics that include the impact of injury-related ischemia / prognostic consideration.

## Neurovascular Colocalization and Coordination

Blood vessels and peripheral nerves are often found running parallel to each other in the body (16, 17), share many analogous mechanisms during development and regrowth (18), and serve as structural guidance cues during regeneration, as blood vessels can direct neurite outgrowth in the absence of intact basal lamina tubes (19). The branching, arborized similarities in structure between nerves and blood vessels were first noted by Vesalius in 1543 (18), and the first study of the blood supply of peripheral nerves was performed in 1768 (11). Details of vascular supply to nerves were expanded by Ramon y Cajal in 1890 (20), and further descriptions and characterizations were published by Adams (1942) (21, 22) Sunderland (1945) (23), Blunt (1957) (24), and Lundborg (1975) (11).

**Figure 1:**
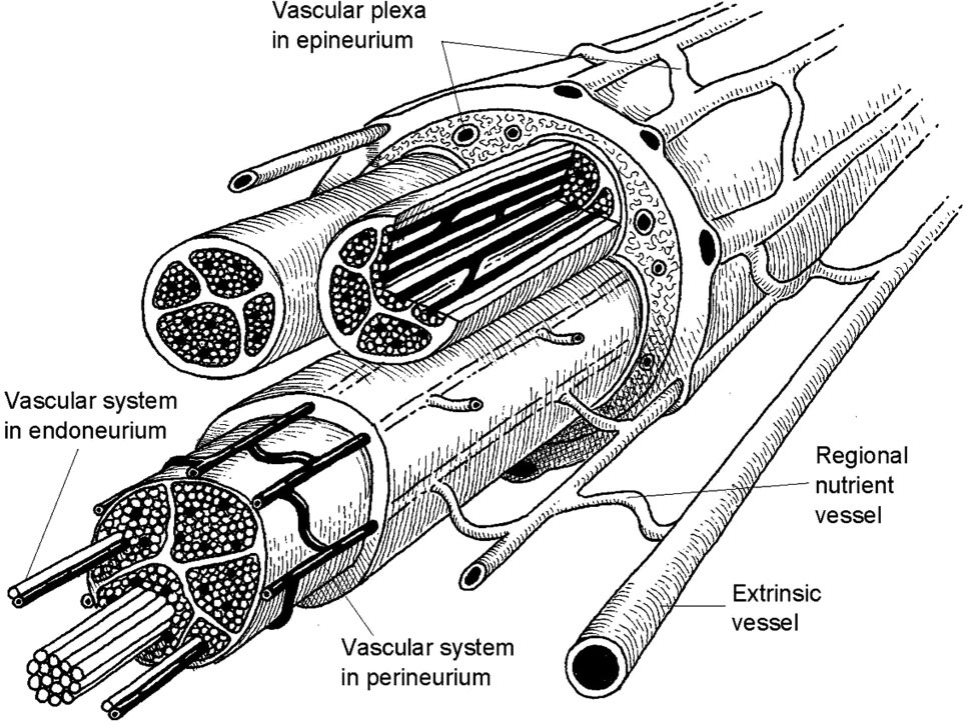
Blood supply in peripheral nerves (Reprinted from Lundborg, G: *Nerve Injury and Repair,* New York, page 43, 1988) (25).

Because of their high metabolic demands, peripheral nerves are highly vascularized tissue, with two separate but interconnected supply networks: intrinsic and extrinsic vessels (Figure 1) (11). The extrinsic supply originates from nearby large arteries and veins in adjacent tissue, which connect / the intraneural intrinsic system via coiled, tortuous vessels that allow relative movement and stretching of the nerve (7, 26). The intrinsic system is comprised of the arterioles and venules of the epineurial, perineurial, and endoneurial plexuses that run longitudinally along the nerve, along with perpendicular communicating vessels, anastomoses, and arteriovenular shunts (11, 13). These highly interconnected and redundant networks have each been shown to provide adequate supply to maintain nerve function, even when the alternate system is completely ligated (11, 22, 27, 28). Although nerves are resilient to ischemia, sufficient mobilization or disruption of the extrinsic system along the length of the nerve has been shown to cause ischemic injury. Lundborg (11) showed that mobilization of 15 cm of intact rabbit sciatic nerve was enough to impair intraneural microcirculation, and that with additional disruption of the intrinsic system via transection this critical distance dropped to 7 cm; highlighting the importance of perfusion in nerve regeneration.

## Perfusion and Ischemia in Nerve Injury

The importance of preservation of blood supply has been known since the 1940s, when cable grafts, which use multiple small donor nerves, or pedicled nerve grafts, where nerves are transferred in stages to maintain blood flow, were used to combat central necrosis seen when using medium to large caliber nerve grafts (29). The resumption of perfusion in nerve autografts relies on both the proximal and distal stumps as well as the surrounding tissue which occur at differential rates (30). However, the relative importance of each component to graft survival, and whether the primary mechanism for vascular integration is centripetal neovascularization or inosculation from surrounding tissue, remains controversial (31). By the 1970s, free vascularized nerve grafts were performed (32), and subsequent studies showed improved or inconclusive recovery of vascularized grafts vs. nonvascularized grafts (29, 33). Unfortunately, the increased technical challenge and the risk of thrombosis in vascularized nerve grafts (34) have limited their application to large defects or complex cases. Proximal nerve lesions such as brachial plexus injuries were another common target for vascularized nerve graft (35); however, direct measurement of efficacy has been inconclusive because of heterogeneity in case application and outcome measures.

Ischemia has been implicated as an important mechanism leading to failed regeneration after nerve trauma, primarily as a promoter of a proinflammatory environment through cellular necrosis, macrophage activation and polarization, and endothelial upregulation of adhesive molecules, such as ICAMs and selectins, that recruit circulating leukocytes and lead to further inflammation and cell death (36, 37). Experimental models of nerve ischemia have required extensive upstream ligation (12, 13) to outweigh distal extrinsic perfusion, and models using whole-limb pressure cuffs have noted that even with six hours of constriction, blood flow returns within minutes (38). In response to ischemic conditions, vasodilators such as calcitonin gene-related peptide and endothelial nitric oxide synthase accumulate, leading to hyperemic conditions immediately after reperfusion (39). Hyperemia has also been directly measured after nerve constriction, crush, and transection injuries (13, 38, 40). Extended periods of ischemia causes damage to endothelial cells, resulting in a long-term reduction in perfusion, termed “no-reflow,” which has been seen after three (12) to eight (38) hours of vessel compression. These numerous pathophysiologic responses lead to a complex temporal profile of neural blood flow after injury, and this complexity of mechanisms is reflected by the varied and conflicting reports published on the role of neurovascular ischemia.

Stretch (41, 42) and compression (43–46) injury models have been used to examine the impact of blood flow on nerve function. Transient nerve compression of 50 mmHg reduces nerve conduction velocity and increases epineurial vascular permeability (47), while more severe crush models using clamps or forceps create a focal injury that can result in axonal degeneration. Blood flow measurements of crush-injured nerves found a 30% reduction in perfusion 24 h after crush injury, followed by significant hyperemia at 48 h after injury (48). Nerve stretch results in acute reduction in blood flow beginning at 8% elongation, with complete cessation of flow around 15% (49). When the nerve is relaxed hyperemia occurs, although some vessels may remain occluded or suffer microthrombi and emboli. Surgical repair under tension causes significant reduction in neurite outgrowth (50), and repair under tension demonstrates worse outcomes than low-tension repair with autograft, despite two neurorrhaphy and suture sites (51). Ischemia, along with repair site failure, may be part of the explanation. Unsurprisingly, experiments that involved supplying nerves with hyperbaric oxygen removed the conduction block produced at high strain conditions (41).

**Figure 2:**
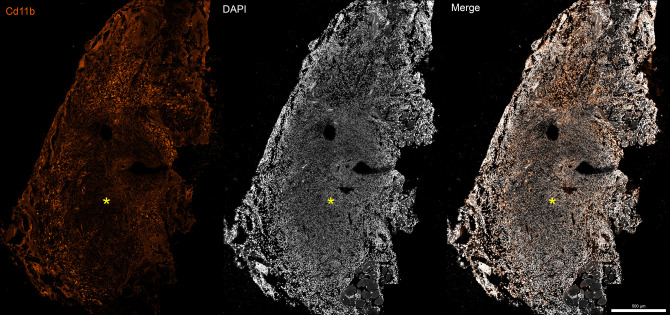
Confocal image (100×) of neuroma-in-continuity formation at the zone of stretch-rupture injury at the hamstring bifurcation, 14 days after injury. Necrotic core (yellow asterisk) is visualized by nuclear disintegration, as hallmarked by blurring of nuclear staining (4′,6-diamidino-2-phenylindole). Necrosis is further demonstrated by large aggregation of CD11b+ cells (orange), a pan-granulocyte marker, which have been associated with clearance of nonviable cells.

Rapid nerve stretch experiments (52, 53) have observed that stretch injury first disrupts the epineurium, the primary anastomotic vascular layer, at lower strain values than the endoneurium, a finding that contradicts Sunderland’s injury grades. At lower stretch severity, intraneural blood vessels remain intact, and the nerve is not ischemic despite the loss of the epineurium. When the nerve is ruptured and intraneural vessels are torn, histologic evidence indicative of necrosis ensues (Figure 2). This is consistent with Lundborg’s findings: endoneurial perfusion appears to be more crucial than epineurial. Furthermore, necrosis leads to a worsened regenerative fate, as discussed above in relationship to nerve grafting.

## Dual Influence of Hypoxia and Vasculature on Nerve Regeneration

In addition to providing oxygen and nutrients to regenerating tissue, nerve vasculature may also provide crucial structural guidance for regenerating axons across damaged tissue and gaps. The process of axonal regeneration has been described in detail, involving the coordinated action of neurites, Schwann cells, and fibroblasts. Nerve regeneration begins with formation of a longitudinally aligned fibrin cable or bridge by day 2 that unites nerve discontinuities (54, 55). More recently, it has been identified that the processes that coordinate Schwann cell migration and neurite guidance depend partially on hypoxia-driven vascular growth as scaffolding in areas of nerve disruption (19).

**Figure 3:**
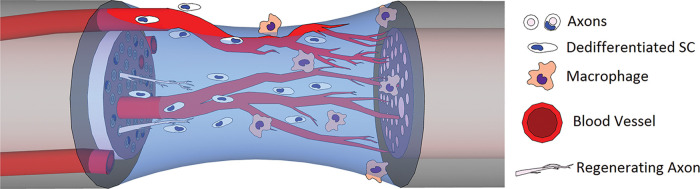
Hypoxia-driven macrophage infiltration / the fibrin bridge leads to polarized angiogenesis via VEGF and other growth factor expression, which then guides Schwann cell migration and neurite outgrowth.

Histological examination of the fibrin bridge reveals a mixture of inflammatory cells (50% macrophages, 24% neutrophils, 13% fibroblasts) and disorganized extracellular matrix (19, 56, 57), presenting an adverse environment for directional migration and growth. Further investigation of the bridge environment revealed that macrophages act as hypoxia sensors through hypoxia-inducible factor-1α (HIF-1α) stabilization, which results in vascular endothelial growth factor-A (VEGF-A) upregulation that initiates polarized angiogenesis / the bridge volume (19). These vessels guide Schwann cell migration / the bridge, with axonal growth cones following the Schwann cells (Figure 3). VEGF has been shown independently to be a major driver of both angiogenesis and nerve regeneration (58, 59). Blood vessels fully infiltrate the bridge by day 3 when the fraction of hypoxic cells drops to less than 10%; while Schwann cells are still fragmenting their myelin sheaths, proliferating, and starting to migrate (60, 61). Artificial misdirection of blood vessel growth via exogenous VEGF-A results in misdirection of axonal regrowth, showing that vasculature alone is enough to independently control nerve guidance (19). Additionally, inhibition of VEGF-A via cabozantinib, as well as knockout VEGF-A models, shows a total lack of Schwann cell and axonal migration / the bridge (62).

Other studies have found similar results in different models. Ablation of HIF-1α slowed nerve regeneration and eliminated the conditioning injury effect (63). Another study simulated increased hypoxia in nerves by targeting its inhibitory molecules prolyl hydroxylase 1–3 (PHD1-3), showing improved axonal outgrowth and nerve regeneration in PHD-knockout mice in response to stimulated angiogenesis (64). VEGF has been well established to improve vascularization, Schwann cell migration, and functional recovery in nerve repair allografts (65–68). Novel treatments for nerve injury have utilized VEGF combined with additional growth factors, including insulin-like growth factor-1 (IGF-1) (69), glial cell–derived neurotrophic factor (GDNF) (70), or nerve growth factor (NGF) (65, 71). Various combinations of nerve regeneration constructs have been reported to support improvements in outcomes, including VEGF and brain-derived neurotrophic factor (BDNF) mimetic self-assembling peptide epitopes embedded in hydrogel matrix (72), VEGF and NGF loaded in an emulsion electrospun fibrous scaffold (73), and gel delivery of VEGF and IGF-1 used to promote functional innervation (69).

Macrophage-driven angiogenesis via VEGF secretion highlights an important contribution of vascular dysfunction. Hypoxia is a driver of chronic inflammation that creates secondary nerve injury and increased fibrosis. Hypoxia is a major chemoattractant for circulating neutrophils and macrophages (56, 57), as well as an activator for resident fibroblasts (19) and vascular endothelial cells (74). Recently, cyclic or intermittent hypoxia, similar to the profile of changing perfusion after nerve injury, was shown to promote a proinflammatory tumor necrosis factor-α and interleukin-8/macrophage inflammatory protein-2–producing macrophage phenotype through activation of the Jun kinase/p65 pathway (75). Similar conditions of prolonged inflammation, such as infection, lead to longer healing times, worse scar formation, and reduced tissue function, whereas nerve regeneration / ischemic wound beds such as skin grafts or poorly perfused limbs is limited (76). Other situations where hypoxia may inhibit nerve health include microvascular disease such as that resulting from diabetes mellitus or radiation injury. Another result of ischemic inflammation relevant to nerves is the induction of inflammatory and neuropathic pain, which can negatively impact regeneration. Macrophage-driven inflammation has been investigated in neuropathic pain, with angiotensin II (77), colony-stimulating factor-1 (78), and transient receptor potential ankyrin 1 (79) pathways implicated as potential therapeutic targets.

There is a fundamental duality to hypoxia for nerve regeneration. Physiologic hypoxia-driven macrophage activity and angiogenesis, which guide and promote axonal regrowth, reside in tension with pathophysiologic chronic inflammation resulting from prolonged hypoxic conditions; this fine balance emphasizes the need for any future therapeutic intervention to incorporate appropriate hypoxia and blood flow on a biologically relevant timeline. Additionally, the critical and early role that blood vessels play in successful nerve reinnervation may indicate that blood flow could be used as a diagnostic tool to inform whether surgical intervention will be required, and to shorten the delay between injury and repair.

## Measuring Blood Flow

Because perfusion is critical to successful nerve regeneration after injury, it should be considered as a potential biomarker of either nerve injury severity or early regenerative success after nerve repair. One challenge for assessment is the absence of a gold-standard measurement technique to determine blood flow in the nervous system. Studies have used a variety of techniques (Table 1), including intravital microscopy (49), hydrogen clearance (HC) (13, 80–82), laser Doppler flowmetry (42, 48, 83–85), ultrasound (86–88), and numerous magnetic resonance imaging (MRI) sequences, including arterial spin labeling (89, 90), dynamic contrast enhancement (DCE) (91, 92), phase contrast (93, 94), blood oxygenation level–dependent (BOLD) (95, 96), and intravoxel incoherent motion (IVIM) (97). HC measures hydrogen gas washout via electrode probe, whereas laser Doppler and ultrasound work by measuring frequency shift from reflected light or sound from moving red blood cells; these all require close proximity or direct contact of the probe head with the nerve. These techniques all have different advantages and disadvantages, but an ideal diagnostic technique should not be invasive (eliminating intravital microscopy, laser Doppler) or terminal (HC), leaving ultrasound and MRI as potential options. Ultrasound is complementary to electrodiagnostic studies, but has reduced resolution for deeply located nerves and is highly operator dependent (98).

**Table 1:** Partial list of blood flow measurement techniques.

Method	Advantages	Disadvantages
HC	Quantitative	Terminal
Laser Doppler	Spatially sensitive	Invasive
Ultrasound	Ubiquitous, noninvasive	Operator dependent, depth limited
MRI-ASL	Endogenous contrast	Low signal to noise, resolution
MRI-DCE	Higher resolution, time specificity	Injected contrast agent, single measurement
MRI-PC	Measures velocity	Unknown
MRI-BOLD	Measures blood oxygenation	Unknown
MRI-IVIM	Measures perfusion	Unknown

**Figure 4:**
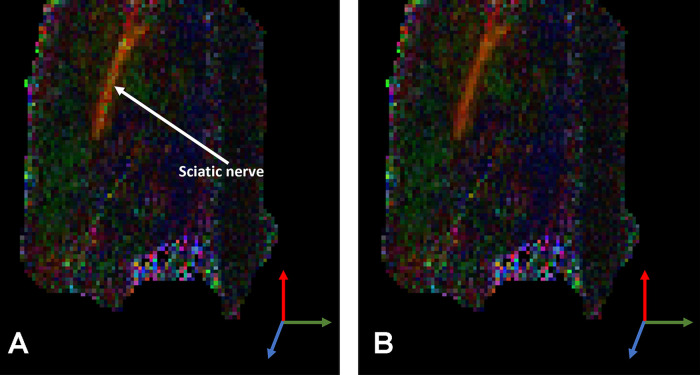
In-vivo primary eigenvector RGB component colormaps weighted by fractional anisotropy (FA) for (**A**) Dfast and (**B**) Dslow tensors in the sciatic nerve of a rat. Mean diffusivities of 2.2 × 10^−3^ mm^2^/s and 1.0 × 10^−3^ mm^2^/s, respectively, indicates blood flow along the longitudinal direction of the nerve.

MRI multiplanar T1, fat-suppressed T2, and diffusion tensor imaging (DTI) sequences have shown promise in detecting microstructural nerve features and remodeling (98–100). Fractional anisotropy (FA), a measure resultant from DTI, has been correlated with successful nerve regeneration as well as failure (101). However, FA remains a retrospective evaluation and does not include flow information that may prove to be prognostic. DCE imaging is a well-established technique that offers reasonable resolution that has recently been used to measure increased blood flow after crush injury (102), but it requires an injected contrast agent and can only capture a “first-pass” effect (92, 103). Additionally, gadolinium has limited diffusion through the blood–nerve barrier, which means it will reflect the status of extrinsic blood vessel networks, but not perfusion within the endoneurium. Arterial spin labeling (ASL) uses endogenous water in blood as the tracer, but is limited by reduced signal-to-noise ratio and spatial and temporal resolution (89, 90). Other flow-sensitive methods such as phase contrast, BOLD, and IVIM are available but have rarely been applied to the peripheral nervous system (98). Preliminary evidence (Figure 4) suggests that IVIM, which determines microcirculation from DTI, demonstrates the ability to capture perfusion within nerves (104).

## Integration of Perfusion / Injury Classification

On the basis of the data presented, perfusion should be incorporated as an essential biomarker for nerve injury and regeneration. However, perfusion does not stand independent of the other aspects of nerve injury. Therefore, to factor perfusion / nerve injury assessment, we conceptualize a three-component model for the evaluation of nerve injuries: (1) anatomical microstructure disruption, advancing considerations of Sunderland’s conceptualization; (2) nerve ischemia from injury; and (3) acute and persistent or pathological inflammatory response.

**Figure 5:**
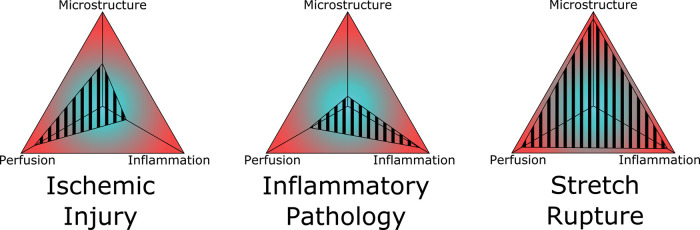
Schematic representation of three-axis nerve injury severity model. Three prototype injuries are shown, comparing primarily ischemic injury, inflammatory injury, and stretch-rupture injury. All three pathophysiologic process are shown to be interconnected, such as a primary perfusion injury impacts inflammation and both have effect upon microstructural remodeling. The more severe the injury on all three axes, the greater the combined area, and thus spontaneous regeneration is less likely.

All three elements are deeply integrated. Mechanical disruption of the nerve leads to loss of endoneurial tube guidance, as well as ischemia from vessel injury. The consequent inflammatory repair microenvironment impacts extracellular matrix remodeling, neurite growth, and angiogenesis. Multidimensional evaluation would potentially allow incorporation and comparison among diverse nerve injury mechanisms, providing a simplified landscape for depicting nerve injuries / one heuristic. For example, principally ischemic injuries, such as positional palsies, versus largely inflammatory injuries, such as amyotrophic neuralgia, can be placed alongside complex, multidimensional injuries such as rapid-stretch injuries (Figure 5). In clinical injuries, all three components are inter-related, as exemplified by fascicular torsion in inflammatory nerve palsies where severe inflammation leads to local ischemia and derangement of nerve microstructure (105). In a three-axis model, the worsening along one axis increases the area of a triangle created with the other two axes—mathematically representing the negative synergistic effect.

Appropriate linked metrics and component weighting will require significant further elucidation. Specifically, our suggested three-axis evaluation could be achieved in clinical practice through:

1. Assessment of microstructural disruption using DTI/FA measurements.
2. Blood flow measurements via IVIM or DCE imaging.
3. A peripheral nerve injury–specific biomarker panel; measured by blood draw, tissue biopsy, or tagged-tracer imaging.

DTI allows for measurements of tissue anisotropy and thus axon and nerve fiber physical integrity, while both IVIM and DCE imaging provide instantaneous information on perfusion and blood flow in the nerve. Biomarker assays would examine evidence of nerve-specific structural proteins or transcripts such as: myelin basic protein, neurofilament, or tau; inflammatory molecules such as IL6 or TNFα, nerve regeneration associated cytokines such as NGF, BDNF, or GDNF; or other nervous system related molecules such as S100, peripherin, or PMP22 (8, 106, 107). We suspect that biofluids will provide meaningful evidence of nerve injury severity or pathophysiologic regeneration, in the same manner that cancer diagnostics are being aided by assessment of extracellular RNA and DNA (108). Either proteomic or transcriptomic approaches may help identify signatures of nerve tissue breakdown, which may better characterize both the severity as well as the consequent recovery potential.

These enhanced metrics may have the potential to predict peripheral nerve injury outcomes and would be of use in assessing the contribution of diseases of microcirculation and inflammation, such as diabetes mellitus, which have profound impact on nerve regeneration. To this end, advances in imaging to identify physiologic process within the nerve in addition to microstructure should be developed concurrently. This multicomponent, diagnostically accurate, prognostically impactful evaluation would then allow optimal decision making, rather than a ‘wait-and-see’ approach that has surrounded nerve injuries for the past two centuries.

## Conclusion

Peripheral nerve injury is a significant and compelling health challenge that would benefit from pathophysiologic-based injury classification. Blood flow is a compelling component of both nerve injury mechanisms and regenerative potential. Peripheral nerve injuries directly impact blood flow within the nerve, and re-establishing vascular networks is critical in nerve regeneration. A classification system that assesses the pathophysiologic components of nerve injury may help better establish peripheral nerve injury severity and its consequent management.
